# Lymph node reactivity and microvessel density in neck metastases of unknown primary squamous cell carcinoma

**DOI:** 10.1016/S1808-8694(15)30973-3

**Published:** 2015-10-19

**Authors:** Ali Amar, Allan Fernando Giovanini, Marilene Paladino Rosas, Onivaldo Cervantes

**Affiliations:** a PhD, Assistant physician – Heliópolis Hospital; b PhD, Positivo University Center; c MS. Pathologist – Heliópolis Hospital; d Associate Professor – Head of the Head and Neck Department – Paulista School of Medicine. Head and Neck Surgery Department - UNIFESP-EPM Head and Neck Surgery Department – Heliópolis Hospital

**Keywords:** squamous cell cancer, lymph nodes, head and neck neoplasms, unknown primary neoplasms, pathologic neovascularization

## Abstract

**Background:**

neoangiogenesis and the immune response are important mechanisms in metastasis development.

**Aim:**

to evaluate lymph node reactivity and microvessel density in neck metastasis of occult primary squamous cell carcinoma considering their histological and clinical variables.

**Study design:**

retrospesctive case-series.

**Method:**

19 patients with neck metastasis of occult primary squamous cell carcinoma who underwent neck dissection between 1983 and 2000 were selected. The lymph nodes were reevaluated on the type of reactivity in both the cortical and paracortical areas, and the metastasis were assessed as to grade, desmoplasia, necrosis and microvessel density (CD34). The relationship between histological and clinical variables was evaluated.

**Results:**

the median microvessel density was 91 vessels/mm2, varying from 28 to 145. Paracortical hyperplasia was more common in patients below 55 years of age (90% x 44%, p=0.05), but there was no relationship between reactivity patterns and microvessel density with prognosis. The disease-free survival was 52% in 3 years, being similar in both groups, with higher or lower microvessel densities.

**Conclusion:**

microvessel density in neck metastasis of occult primary squamous cell carcinoma had a great individual variability. It wasn't possible to establish the relationship between microvessel density and the clinical or histological variables studied.

## INTRODUCTION

Neoangiogenesis is an important process for the development of tumors and is related with metastasization and the prognosis in different types of malignant neoplasms^1^, ^2^, ^3^, ^4^. The immune response, as well as anti-tumor action, is part of the modulation of angiogenesis and other related mechanisms to the invasion and growth of the neoplasms. Regional lymph nodes frequently present signs of reactivity, the meaning of which is still unknown^5^, ^6^. This study aims to evaluate microvascular density and linfonodal reactivity in cervical metastases of occult primary tumors, relating these findings to other histological and clinical variables, considering that this disease is an adequate clinical model for the study of metastases.

## METHODS

All patients that had tissue samples available for analysis and follow-up of at least 12 months following initial treatment were included totalling 19 cases. Of these 16 patients were male and 3 were female. The average age was 55 years. Smoking and alcohol abuse were present in 16 and 13 cases respectively. Patients were staged according to the TNM 2002 classification (UICC-AJCC); 1 patient was stage N1, 8 patients were stage N2, and 10 patients were stage N3. The average number of nodes removed during radical neck dissection was 22.4 lymph nodes, varying from 4 to 49. Reactivity was evaluated in 427 lymph nodes and microvascular density was assessed in 60 lymph nodes with metastases. Conventional histological analysis evaluated the degree of differentiation, the presence of tumor necrosis, desmoplasia and lymph node reactivity. Lymph node assessment used Berlinger et al.'s and Klimek et al.'s criteria, as follows^5^, ^7^:

A - Paracortical hyperplasia: increased paracortical celularity.
B - Follicular hyperplasia: increased number and size of lymphoid follicles in the lymph node cortical zone, formation of secondary follicles with prominent germinative centers.
C - Sinus hyperplasia: endothelial cell hyperplasia with medullary sinusoidal distension and histiocitosis.
D - Non-stimulated lymph node: lymph nodes with no lymphocyte proliferation.
E - Depleted lymph node: decreased lymphocyte population and diffuse fibrose in lymph nodes.

Reactivity was classified according to Amar et al., evaluating cortical and paracortical lymph node zones, taking into account the pattern in more than 2/3 of reactive lymph nodes. With no predominance, hyperplasia was considered as mixed. Lack of reactivity in more than 80% of lymph nodes defined the non-reactive pattern. Sinusal hyperplasia and histiocitosis were assessed separately. Histiocitosis was defined as the presence of macrophages in sinusoids, characterized by large nuclei, evident nucleoli and eosinophilic cytoplasm. Conventional histological analysis included the degree of differentiation, the presence of tumor necrosis and desmoplasia. Immunohistochemical reactions were made in the following order:

1. Primary antibody (Monoclonal Mouse Anti-Human CD34 Class II, Dako, M7165, Denmark) diluted at 1:100 in PBS, for 18 hours at 4°C in a humid chamber.
2 Secondary antibody (Biotinylated Goat Anti-Mouse/Rabbit Ig, of the StreptAB Complex/HRP Duet Mouse/Rabbit, Dako, K492, Denmark) diluted at 1:200, for 30 minutes at 37°C.
3 Reagent complex A (Streptavidin) and reagent B (Biotinylated Peroxydase) diluted at 1:200, for 30 minutes at 37°C.
4 Solution of 3,3’-Diaminobenzidine Tetrahydrochloride 60 mg% (Sigma, D5637, USA), 1 mL of Dimethylsulphoxide (DMSO), 1mL of 100 H2O2 at 6% and mL of PBS, for 5 minutes at 37°C, in the dark.

Microvessel counting was done under optic microscopy in 5 areas of greater intra-tumor vascular density (hot spots). A 0,4 mm2 area in each hot spot was selected using the image analysis program Image Tool 2.0 (University of Texas Health Science Center, USA); the number of microvessels were counted at 100x magnification and the values obtained in the 5 areas (2 mm2) were added. The relation between lymph node reactivity and metastatic microvascular density, the degree of differentiation, desmoplasia, necrosis, clinical findings and progression of patients were assessed.

The Kaplan-Meier actuarial survival method was used for statistical analysis of and the differences between the groups were assessed using Gehan's test. For quantitative variables, Mann-Whitney's U test and the Kruskal-Wallis test were used to assess differences between the groups. Correlation between quantitative variable was done with Spearman's test. For qualitative variables, Fisher's exact test and the difference between two variables test were used, taking into account bicaudal values for p. Calculation was done with the software Statistica 5.1 (Statsoft Incorporation, USA) and differences in all tests were considered significant when p-values were equal or below 0.05.

## RESULTS

Reactivity was assessed in 427 lymph nodes, of which 102 presented paracortical hyperplasia and 122, follicular hyperplasia. Sinusal hyperplasia was observed in 93 lymph nodes from 15 patients, which also presented sinusal histiocitosis. Paracortical hyperplasia, including its presence mixed pattern cases, was seen in 90% of patients below age 55 and in 44% of patients aged 55 and above (p=0.05).

Average microvascular density was 91.5 microvessels/mm2, varying from 28 to 145. Considering independently the degree of differentiation in each lymph node and the respective microvascular density, well differentiated or moderately differentiated tumors had a mean 55 microvessels/mm2 (Q25-75% = 38-90), whereas undifferentiated tumors had a mean 53 microvessels/mm2 (Q25-75% = 43-116), p=0.85.

On follow-up, primary tumors was diagnosed in 3 patients, neck recurrences were diagnosed in 10 patients and 4 cases had metastases at a distance. In total, 5 patients received salvage treatment, of which 4 underwent surgery and 1 was treated with radiotherapy. The remaining patients received palliative treatment. Of patients undergoing salvage treatment, 3 patients had the disease controlled in the second treatment.

The mean microvascular density in cases that had metastases at a distance was 73 microvessels/mm2 (Q25-75% = 55-108) and the mean microvascular density in cases that did not have metastases at a distance was 99 microvessels/mm2 (Q25-75% = 28-145), p=0.59.

The disease-free survival for the whole group was 52% in 2 years and 44% in 5 years. Asymptomatic patients had an average 61 months follow-up time.

No significant relation was observed between the age of patients and microvascular density of metastases.

## DISCUSSION

The first issue in cases of cervical metastases from occult primary tumors pertains to the origin of the tumor. Patients with occult primary tumors have similar epidemiological characteristics compared to patients with upper digestive tract and upper airway primary tumors. Furthermore, similar genetic alterations were found in so called normal oral cavities and in lymph node metastases in some cases of occult primary tumors that, when analyzed within the context of field cancerization and clonal expansion, are strong evidence supporting the hypothesis of their origin in the upper digestive tract and the upper airway epithelium, with possible regression or tumor dormancy in the primary site^8^, ^9^, ^10^. Many studies report the association between microvascular density in primary tumors and metastases^3^, ^4^.

**Figure 1:**
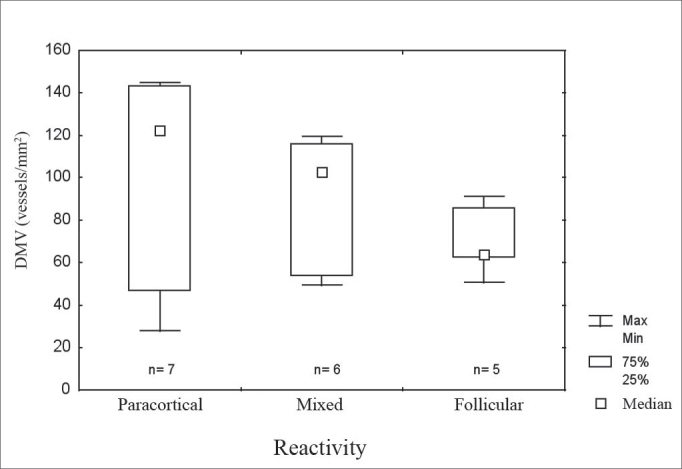
Microvascular density in metastases in relation to the pattern of lymph node reactivity.

legend: DMV = microvascular density. Max = maximum. Min = minimum. 75% = 75% quartile. 25% = 25% quartile. Kruskal-Wallis test, p=0.27.

**Figure 2:**
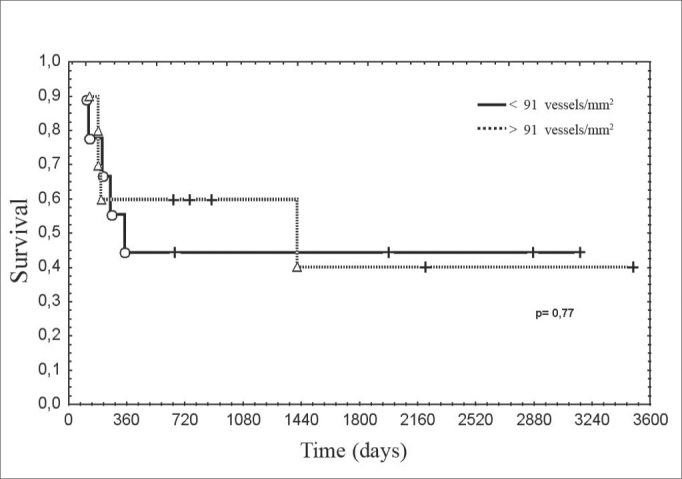
Disease-free survival according to microvascular density in metastases.

legend: Actuarial survival Kaplan-Meier method. Gehan's test, p=0.64

**Figure 3:**
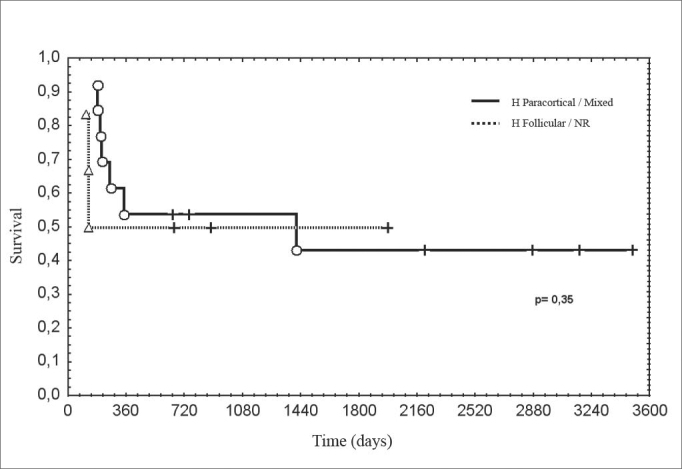
Disease-free survival according to the pattern of lymph node reactivity.

legend: Actuarial survival, Kaplan-Meier method. Gehan's test, p = 0.35.

**Figure 4:**
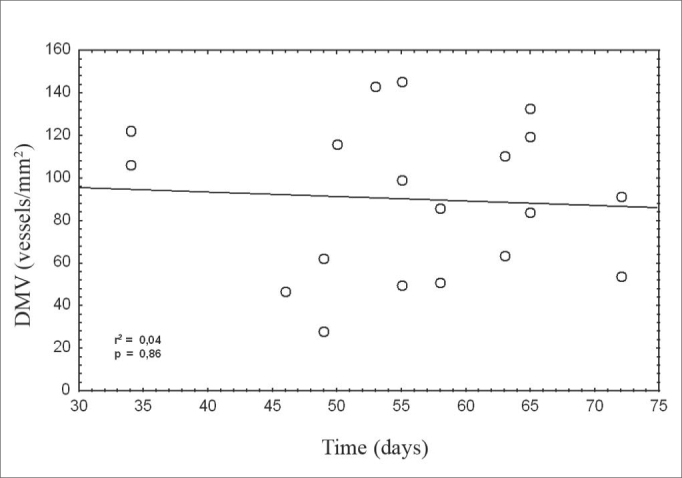
Correlation between the age of patients and microvascular density of metastases.

legend: DMV = microvascular density. Spearman's test, r2 = 0.04, p = 0.86.

As neoangiogenesis is an important feature for the growth of metastases, increased angiogenesis suggests an advantage acquired due to clonal selection. An inverse relation was reported between microvascular density in the primary tumor and its respective metastases, suggesting that increased angiogenesis in a specific site might inhibite angiogenesis at a distance^11^, ^12^. The hypothesis that metastases from occult primary tumors could inhibit the growth of the primary tumor could be supported by increased microvascular density, however we found great variability in our study. This variability does not eliminate the hypothesis that systemic control of neoangiogenesis is implied in the etiogeny of the occult primary tumor, although this may not be the only mechanism involved.

**Table 1:** Microvascular density according to clinical characteristics and histological variables.

Microvascular Density					
		Median Q_25-75%_	Mim	Max	p
Age	≤ 55	102 (49-122)	28	145	0,82
	> 55	86 (63-110)	51	133	
N Stage	N1-2	62 (55-99)	47	145	0,20
	N3	108 (86-122)	28	143	
Reactivity	Paracortical	122 (47-143)	28	145	0,27
	Mixed	102 (53-116)	41	119	
Reactivity	Follicular	63 (62-86)	59	91	0,64
	≤ 50%	91 (49-106)	28	145	
	> 50%	86 (62-119)	51	143	
Evolution	Sick	88 (62-119)	28	145	0,86
	Asymptomatic	99 (53-110)	47	143	
Necrosis	No	86 (53-116)	47	143	0,52
	Yes	102 (84-113)	28	145	
Desmoplasia	No	63 (51-116)	28	122	0,22
	Yes	102 (84-133)	47	145	

A further important issue is the meaning of tumor microvascular density, particularly when using the hot spot count technique^3^, ^13^. The tumor may grow along existing microvessels or remodel the stroma, promoting neoangiogenesis; however, increased microvascular density does not mean that the tumor has an increased blood supply. Necrotic tumors may have increased neoangiogenesis, possibly caused by an increased inflammatory response in this type of cell death^14^. In order to have diffusion of oxygen and nutrients, cells may be up to 110 mm from blood microvessels, a measure that is reached with low microvascular density^13^. Lymph nodes have a high microvascular density, surpassing density observed in metastases; thus the change in blood flow caused by the growth of metastases also reinforces the idea that tumors modify the vascular structure and the dynamics of flow^15^, ^16^. Unfortunately, the technique we used does not allow us to separate neoformed from existing microvessels.

There was no correlation between age and microvascular density, however, paracortical reactivity was found in greater frequency among patients aged below 55 years. As decreased angiogenesis is expected with aging, this finding suggests that neoangiogenesis in metastases is related to increased inhibition^17^.

Lymph node reactivity, seen as paracortical or follicular hyperplasia, was related to the best prognosis in different studies^5^, ^6^, ^18^. In various studies there was no consensus on which type of reactivity has the best prognosis, or a method to classify heterogeneous reactivity. It is not uncommon to find more than one pattern in the same lymph node or different patterns in many lymph nodes in the same patient, which makes it difficult to classify these patients, and which might explain the variety of results. Most patients with occult primary tumors had reactive lymph nodes. The immune response is related to the release of angiogenesis promoting substances. In this study, cases that had paracortical reactivity had the highest microvascular densities, although not statistically significant. In head and neck tumors lymph nodes with follicular or paracortical hyperplasia are located close to the tumor, that is, those that habitually have metastases. Therefore, it is possible that the immune response favors the implantation and the growth of lymph node metastases.7 Furthermore, the immune response could be responsible for primary tumor regression.

Histiocitose and sinusal hyperplasia were found in most patients. The latter is considered as non-specific reactivity and is associate with immunological dysfunction^7^. Being present in most patients, it was impossible to establish its relation with the other variables in the study. Sinusal hyperplasia is more frequent in lymph nodes distant from tumor, so we canot discard the possibility that the tumor or the immune response in the first lymph nodes down the lymph channel may cause an immune supressor effect upstream of the lymphatic flow^7^. This hypothesis is reinforced by the fact that lymphocytes from lymph nodes in the tumor drainage area have a lower cytotoxic action compared to lymphocytes in peripheral blood^19^. Macrophages (histiocytes) have an important function in remodelling the extracellular matrix and in modulating angiogenesis and the immune response. Macrophages can also modify the genic expression and the behavior of tumor cells, increasing their invasive potential. This action could both favor or inhibit tumor growth^20^, ^21^.

Disease-free survival was similar between patients with increased or decreased microvascular density. Studies reporting the highest microvascular density with the less favorable prognosis had evaluated primary tumors^4^. As increased microvascular density is associated with metastases, this might explain the unfavorable progression in these cases. Decreased radiosensitivity has been reported in primary tumors that had extreme microvascular density, which might reflect regional modulation of neoangiogenesis^22^, ^23^. In our study, multimodal treatment may have masked this correlation. Considering the great variation of microvascular density, also found in other studies, the sample was insufficient to establish significant relations with other clinical or histological variables^24^, ^25^.

In conclusion, lymph node reactivity and microvascular density in metastases from occult primary tumors are not correlated and have no relation to disease progression. In these patients, where the effect of the primary tumor on study parameters may be disregarded, it appears that lymph node metastases, one of the most relevant prognostic factors in upper digestive tract and upper airway carcinomas, reflects clonal selection in which neoangiogenesis has minor importance. Furthermore, the classification we used may have masked correlations existing only in the metastatic microenvironment. Microvascular density reflects a form of interaction with adjacent tissues, essential for tumor growth, that may have regional or systemic effects. Obviously, the prognosis of an individual is multifactorial and microvascular density evaluates only a single moment within a fraction of the tumor. We were unable to clarify these functional aspects by the morphologic alterations we analyzed.
